# Arterial Tortuosity and Its Correlation with White Matter Hyperintensities in Acute Ischemic Stroke

**DOI:** 10.1155/2022/4280410

**Published:** 2022-03-24

**Authors:** Ke Shang, Xiao Chen, Chang Cheng, Xiang Luo, Shabei Xu, Wei Wang, Chenchen Liu

**Affiliations:** ^1^Department of Neurology, Tongji Hospital, Tongji Medical College, Huazhong University of Science and Technology, Wuhan, China; ^2^Department of Radiology, Tongji Hospital, Tongji Medical College, Huazhong University of Science and Technology, Wuhan, China

## Abstract

**Introduction:**

The association between arterial tortuosity and acute ischemic stroke (AIS) has been reported, but showing inconsistent results. We hypothesized that tortuosity of extra- and intracranial large arteries might be higher in AIS patients. Furthermore, we explored the correlation between artery tortuosity and white matter hyperintensity (WMH) severity in AIS patients.

**Methods:**

166 AIS patients identified as large artery atherosclerosis, and 83 control subjects were enrolled. All subjects received three-dimensional computed tomography angiography (CTA). Arterial tortuosity was evaluated using the tortuosity index. WMHs were evaluated using magnetic resonance imaging in all AIS patients.

**Results:**

AIS patients showed significantly increased arterial tortuosity index relative to controls, including left carotid artery (CA) (*p* = 0.001), right CA (*p* < 0.001), left common carotid artery (CCA) (*p* < 0.001), right CCA (*p* < 0.001), left internal carotid artery (*p* = 0.001), right internal carotid artery (*p* = 0.01), left extracranial internal carotid artery (EICA) (*p* < 0.001), right EICA (*p* = 0.01), and vertebral artery dominance (VAD) (*p* = 0.001). The tortuosity of all above arteries was associated with the presence of AIS. AIS patients with moderate or severe WMHs had a higher tortuosity index in left CA (*p* = 0.005), left CCA (*p* = 0.003), left EICA (*p* = 0.07), and VAD (*p* = 0.001). In addition, the tortuosity of left EICA and VAD was associated with WMH severity in AIS patients.

**Conclusions:**

Increased extra- and intracranial large arteries tortuosity is associated with AIS. The tortuosity of left carotid artery system and vertebral artery may be the independent risk factors for WMH severity in AIS patients. *Clinical Trial Registration*. This trial is registered with NCT03122002 (http://www.clinicaltrials.gov).

## 1. Introduction

Arterial tortuosity is a common vascular anomaly that frequently occurs in almost all vessels in the body, especially extra- and intracranial arteries [1]. Clinical data suggest that artery tortuosity has been associated with aging, genetic defects, hypertension, and atherosclerosis [2–4]. In clinical practice, tortuosity of cerebral arteries has been frequently reported in patients with acute ischemic stroke (AIS). In recent decades, several studies have been performed on the relationship between arterial tortuosity and ischemic stroke, showing inconsistent results. In few previous studies, carotid artery tortuosity measured by color Doppler ultrasound seems not associated with risk factors of AIS and not related to ischemic stroke or the presence of carotid stenosis [5, 6]. Further studies also suggest that arterial tortuosity may not have a significant association with carotid stenosis or atherosclerotic plaque by using computed tomography angiography (CTA) and digital subtraction angiography (DSA) imaging, respectively [7, 8]. On the contrary, vascular tortuosity has been identified that it may be related to intracranial artery atherosclerosis [9]. Moreover, a recent study showed carotid artery tortuosity is an independent predictor of outcomes for AIS [10]. Therefore, whether arterial tortuosity is linked to AIS remains uncertain.

Cerebral white matter hyperintensities (WMHs) are considered to be main manifestation of cerebral small vessel disease (CSVD) [11]. It has been linked to cognitive decline, gait disturbance, depression, and increased risk of stroke [12]. It is known that advanced age and hypertension are the well-established risk factors related to WMHs [13, 14]. Other risk factors including diabetes mellitus, genetic factors, and homocysteine are also identified [14–16]. Some studies indicated arterial tortuosity may be associated with an increased risk of WMHs in elderly population [17, 18]. However, another research suggest that it is brain vessel density, but not vessel tortuosity, that is related to WMH lesions in young adults [19].

Based on the hypothesis that increased arterial tortuosity might be associated with AIS, we compared the tortuosity of extra- and intracranial arteries in patients with AIS to that of control subjects in this work. Furthermore, we also analyzed the correlation between cerebral arteries tortuosity and severity of WMHs in AIS patients.

## 2. Methods

### 2.1. Study Subjects and Data Collection

This is a single-center, observational case-control trial based on a prospectively collected registry data for patients with acute ischemic stroke, who were admitted to the Department of Neurology of Tongji Hospital between April 2018 and January 2020. Among them, patients with AIS were identified as large artery atherosclerosis according to TOAST classification. All patients underwent CTA and magnetic resonance imaging (MRI). Patients were excluded if they (1) have other etiologies of AIS, such as atrial fibrillation, valvular heart disease, patent foramen ovale, dissection, and moyamoya disease and (2) were unsuitable for the measurement of arterial tortuosity, such as poor CTA imaging quality. Control subjects without previous stroke history who also received CTA imaging in the same time period were identified as the control group (Supplementary Figure 1). The information data for each subject, including demographic characteristics, medical history, and risk factors of stroke, were obtained from the prospectively registered database. Blood samples were drawn from patients within 7 days after stroke onset and control subjects. The result of plasma glucose, glycosylated hemoglobin (HbA1c), total cholesterol, low-density lipoprotein cholesterol (LDL-C), high-density lipoprotein cholesterol (HDL-C), triglyceride, uric acid, serum creatinine, C-reactive protein, fibrinogen, and homocysteine was obtained from the blood sample examinations. Quantification of urine protein was analysis of a 24-hour urine collection. All AIS subjects who had MRI were scored their WMHs by two experience radiologist who were blinded to the clinical data, using the visual rating scale proposed by Fazekas grades ranging from 0 to 3 [20]. Patients were divided into three groups according to the Fazekas grades: none to mild (Fazekas 0-1), moderate (Fazekas 2), and severe (Fazekas 3).

### 2.2. Measurement of Arterial Tortuosity

**Figure 1:**
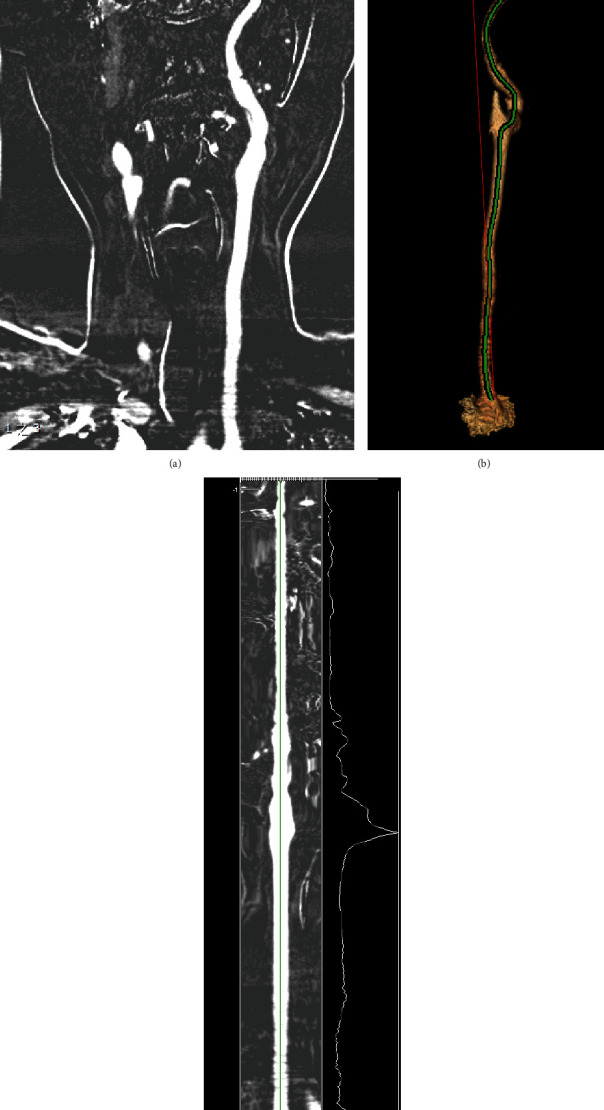
Measurement of arterial tortuosity computed tomography angiography (CTA), illustrated by the example of carotid artery (CA). (a) Left CA in multiplanar reformation. (b) The straight length (red line) and actual length (green line) are measured in three-dimensional reconstructed CTA. (c) The green line represents the actual length of left CA by using curved planar reformat.

Contrast-enhanced CT angiography was performed using Toshiba Aquilion ONE 320 CT scanner. Intravenous contrast medium 370 mg I/mL iopromide (Ultravist 370, Bayer Schering Pharma, Berlin, Germany) was administered at a flow rate of 5-6.5 mL/s, followed by a 30 mL saline flush. The total contrast volume was calculated according to weight. The precise timing of the CT imaging acquisition was determined using a bolus-tracking technique: when the threshold was reached (+90 HU above the baseline in a region of interest drawn on descending segment of aortic arch), the acquisition was performed in a caudocranial direction from the aortic arch to the cranial crest. Acquisition parameters were as follows: slice thickness of 0.5 mm, matrix 512 × 512, and 120 kVp. All original images were transmitted to an imaging workstation (ADW 4.6; GE Medical System, Milwaukee, WI) for processing. A 3D volume-render angiogram was created from the source imaging of CTA for each patient. The arteries in each patient were measured by two experienced radiologists who were blinded to the patient's diagnosis and outcome. By specifying the starting and ending points, the software can automatically track the route and plot a path that follows the tortuous vessel in 3D space. The software allows the user to rotate the 3D volume-rendered image in arbitrary direction. As the 3D image is rotated, the plotted path rotates with the image and can be adjusted in real time, ensuring that the final measured path is the centerline of the arteries. Actual length was measured by tracing the course of arteries from origin to the end. Straight length was measured as the linear distance in 3D space from the origin to the end (Figure 1). The tortuosity was evaluated using tortuosity index, which was defined as (actual length/straight length) − 1 × 100 in each artery [21]. This process was repeated for each of the following extra- and intracranial arteries: bilateral carotid arteries (CA), bilateral common carotid arteries (CCA), bilateral internal carotid arteries (ICA), bilateral extracranial internal carotid arteries (EICA), bilateral intracranial internal carotid artery (IICA), M1 segments of bilateral middle cerebral arteries (M1), vertebral artery dominance (VAD), and basilar artery (BA). Each vessel by location is detailed in Supplementary Figure 2.

### 2.3. Statistical Analysis

**Figure 2:**
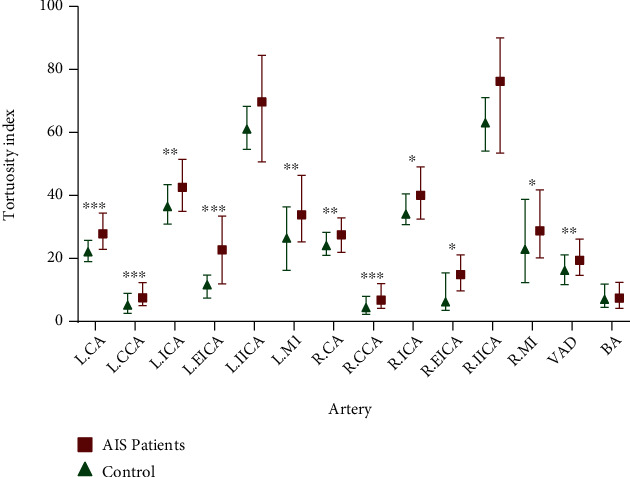
Forest plots show correlation analysis of arterial tortuosity with WMH severity in AIS patients. The adjusted odd ratio and *P* value represent the results of multivariate logistic regression analysis. Variables entered into analysis includes age, male, and hypertension. ∗*P* < 0.05.

All data were analyzed using SPSS23.0 software (IBM Corp., Armonk, NY, USA). Data were presented as median ± interquartile (IQR) or number of subjects (%). Pearson's chi-square test was used for univariate analysis of categorical variables. The one-way analysis of variance was used for univariate analysis of continuous variables. The Mann-Whitney *U*-test was used for comparisons of variables without homoscedasticity. Multivariate analysis was performed using logistic regression to investigate the relationship of tortuosity index with ischemic stroke and WMHs, respectively. In Table 1, the multivariate analysis was adjusted for age, sex, hypertension, diabetes mellitus, hyperlipidemia, current smoking, alcohol abuse, LDL-C, C-reactive protein, homocysteine, fibrinogen, and proteinuria. In Figure 2, the multivariate analysis was adjusted for age, sex, and hypertension. The results were expressed as crude/adjusted odds ratios (OR) and their 95% confidence intervals (CI). *P* value < 0.05 was assumed to indicate a statistically significant association.

## 3. Results

### 3.1. Baseline Characteristics

A total of 166 AIS patients (115 males and 51 females) with a mean age of 56.98 ± 0.83 years and 83 control subjects (56 males and 27 females) with a mean age of 55.86 ± 1.28 years were enrolled. There was no significant difference in age (*p* = 0.447) or sex (*p* = 0.772) between the two groups. The demographic data, stroke risk factors, and clinical features of the subjects are described in Table 2. There was a significant difference between the groups in respect of medical history of hypertension (*p* = 0.005), CRP (*p* < 0.001), homocysteine (*p* = 0.002), fibrinogen (*p* = 0.016), and proteinuria (*p* = 0.01). Furthermore, the severity of WMHs was significantly greater in AIS patients (*p* = 0.003).

### 3.2. Correlation between Arterial Tortuosity and AIS

**Figure 3:**
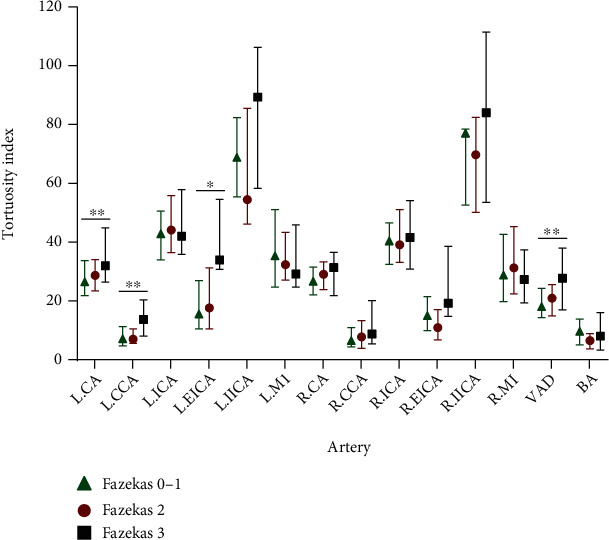
The arterial tortuosity by location in AIS patients and control subjects. AIS: acute ischemic stroke; BA: basilar artery; CA: carotid artery; CCA: common carotid artery; EICA: extracranial internal carotid artery; ICA: internal carotid artery; IICA: intracranial internal carotid artery; L: left; M1: first segment of middle cerebral artery; R: right; VAD, vertebral artery dominance. ∗*P* < 0.05 (AIS patients versus control); ∗∗*P* < 0.01; and ∗∗∗*P* < 0.001.

Compared with control subjects, tortuosity index of arteries was higher in AIS patients: left CA (*p* < 0.001), right CA (*p* = 0.001), left CCA (*p* < 0.001), right CCA (*p* < 0.001), left ICA (*p* = 0.001), right ICA (*p* = 0.01), left EICA (*p* < 0.001), right EICA (*p* = 0.01), left M1 (*p* = 0.001), right M1 (*p* = 0.032), and VAD (*p* = 0.001) (Figure 3 and Supplementary Table 1). There was no statistically significant difference between the groups in terms of left IICA, right IICA, and BA.

By multivariate logistic regression analysis, tortuosity index of the left CA (OR, 1.145, 95% CI, 1.068-1.217; *p* < 0.001), right CA (OR, 1.059, 95% CI, 1.007-1.113; *p* = 0.025), left CCA (OR, 1.106, 95% CI, 1.031-1.187; *p* = 0.005), right CCA (OR, 1.066, 95% CI, 1.01-1.126; *p* = 0.02), left ICA (OR, 1.044, 95% CI, 1-1.098; *p* = 0.048), left EICA (OR, 1.307, 95% CI, 1.037-1.646; *p* = 0.023), left IICA (OR, 1.077, 95% CI, 1.014-1.143; *p* = 0.016), right IICA (OR, 1.067, 95% CI, 1.012-1.124; *p* = 0.015), left M1 (OR, 1.026, 95% CI, 1.001-1.052; *p* = 0.043), right M1 (OR, 1.029, 95% CI, 1.006-1.053; *p* = 0.015), and VAD (OR, 1.06, 95% CI, 1.009-1.113; *p* = 0.021) was associated with the presence of AIS (Table 1). The receiver operating characteristic (ROC) curve of arterial tortuosity for predicting AIS is shown in Table 1. The areas under the ROC curve (ACU) were 0.79 (0.664-0.917) for left EICA, which have the best value.

### 3.3. Correlation between Arterial Tortuosity and WMHs in AIS Patients

**Figure 4:**
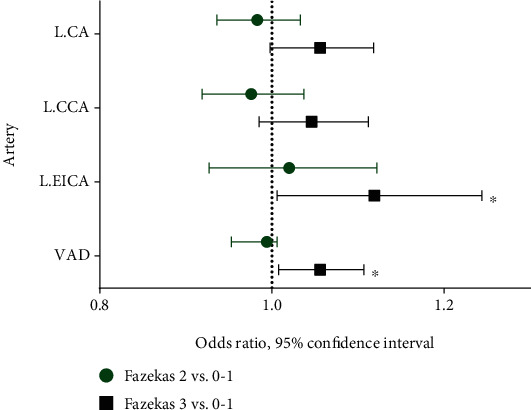
The arterial tortuosity by location in severity of WMHs in AIS patients. ∗*P* < 0.05 and ∗∗*P* < 0.01.

Of the 166 AIS patients, baseline demographic and clinical characteristics by WMH severity were provided in Supplementary Table 2, and there was a significant difference between the groups in age (*p* < 0.001). When compared with patients with none and mild WMHs, patients with moderate or severe WMHs had a greater arterial tortuosity in left CA (*p* = 0.005), left CCA (*p* = 0.003), left EICA (*p* = 0.07), and VAD (*p* = 0.001) (Figure 4 and Supplementary Table 3). Logistic regression analysis showed that increased tortuosity of above arteries was not correlated with the moderate WMHs. However, arterial tortuosity of the left EICA (OR, 1.119, 95% CI, 1.006-1.244; *p* = 0.039) and VAD (OR, 1.056, 95% CI, 1.008-1.107; *p* = 0.022) was associated with the presence of severe WMHs after adjusting for age, male, and hypertension (Figure 2 and Supplementary Table 4).

## 4. Discussion

Previous study reported that arterial tortuosity was identified as a result of alterations in embryonic dysplasia but not vascular remodeling secondary to risk factors of AIS [5]. In contrast, mounting studies showed that the tortuosity of arteries significantly increased with age and might be associated with stroke risk factors [4, 9, 22]. Arterial tortuosity syndrome (ATS), which is characterized by widespread elongation and tortuosity of the aorta and mid-sized arteries as well as focal stenosis of segments of the pulmonary arteries and/or aorta, has been found to be associated with mutations in the SLC2A10 gene, which encodes the facilitative glucose transporter GLUT10 [23, 24]. Apart from these inescapable and hereditary conditions, arterial tortuosity was also thought to be associated with intracranial artery atherosclerosis and cervical arterial dissection, as well as pediatric stroke [9, 21, 25–27]. Based on the various causes for AIS, this study only included patients with AIS who were identified as large artery atherosclerosis and excluded cardiogenic emboli, vasculitis, moyamoya disease, arterial dissection, and other causes of AIS. In this case-control study, the tortuosity of extra- and intracranial arteries was higher in patients with AIS than controls. Our study also showed that the tortuosity of these arteries was associated with AIS.

Measurement of arterial tortuosity has been proved to be of clinical utility in identifying diseased vascular. Early studies mainly used Doppler ultrasound to evaluate arterial tortuosity [5, 6]. With the rapid development of neuroimaging techniques, arterial tortuosity is defined on angiography, which is considered to be more precise in general. Moreover, there is no consensus on the classification of arterial tortuosity. Weibel and Fields classified artery abnormality into three types: tortuosity as a C- or S-shaped elongation, coiling, and kinking [28]. Kinking also can be classified according to the severity of the angle between the two segments forming the kink: the angle less than 90° (type 1) or 60° (type 2) or 30° (type 3) [29]. Although these methods can easily reflect the arterial tortuosity, it is unable to accurately measure and quantify arterial tortuosity. In emerging studies, tortuosity index (TI) exists to quantify arterial tortuosity due to its good reproducibility values [21, 30–32]. Therefore, we used the tortuosity index as an objective indicator instead of an artificial judgment based on arterial images for analysis in this study, which can avoid bias and error caused by subjective way.

It is still controversial whether arterial tortuosity is related to AIS. Several studies showed that arterial tortuosity could lead to vertigo, syncope, tinnitus synchronous to pulse, and TIA or ischemic stroke [33, 34]. Besides, patients with TIA or AIS caused by severe arterial tortuosity could benefit from surgical treatment [33, 35, 36]. Our results showed that the tortuosity of majority extra- and intracranial arteries was significantly higher in AIS patients than that of controls. Multivariate logistic regression analysis also confirmed that the tortuosity of these arteries was associated with AIS. Furthermore, we thought tortuosity of artery, especially left EICA, could robustly identify AIS patients, offering good predictive values for distinguishing AIS. The most possible mechanism of the increased risk of AIS may be the hemodynamic alteration caused by arterial tortuosity. A higher prevalence of hemodynamic changes was found in patients with severe arterial tortuosity [3]. Arterial tortuosity will cause the blood pressure drop, which will increase with the severity of arterial tortuosity [37]. Due to adequate cerebral blood flow via the self-regulatory mechanism, the pressure drop may not lead to AIS under physiological conditions. AIS will occur when the self-regulatory mechanism is decompensation due to stroke risk factors such as hypertension, old age, or atherosclerosis [37]. This can explain why not all subjects with abnormal arterial tortuosity develop ischemic stroke. In addition to the hemodynamic changes, arterial tortuosity may also cause luminal stenosis [38], but some studies suggest that the degree of artery stenosis severity was not related to the presence of kinking and coiling of arteries [8]. We suggest that tortuosity index may be a better imaging biomarker used for future research about association between arterial tortuosity and artery stenosis.

The extra- and intracranial large arteries, as the upstream vascular of cerebral small vessels, were thought to be associated with CSVD [39]. The structural changes in these large arteries may change the hemodynamics of cerebral small vessels. In general, WMH is used as an MRI marker to evaluate the severity of CSVD. There exist several evidences that support the association between arterial morphological variations and WMH severity. Some studies revealed that arterial dolichoectasia in stroke patients was independently associated with WMHs [40–42]. The prevalence of carotid artery kinking was significantly higher in WMHs patients, and kinking may be a risk factor of WMHs [18, 43]. Moreover, recent studies established an association between WMHs and arterial tortuosity which was evaluated using the tortuosity index [17, 44]. However, these studies excluded the patients with stroke and only concerned internal carotid arteries tortuosity. To our knowledge, there is no clinical report on the correlation between arterial tortuosity identified as tortuosity index and WMH severity in stroke patients. We reported that tortuosity indexes of left EICA and VAD were associated with the severity of WMHs in AIS patients. Arterial tortuosity causes WMHs through various possible mechanisms. First, increased arterial tortuosity promotes hemodynamic variation in cerebral blood flow which can result in hypoperfusion and chronic brain ischemia and then may increase the risk of developing WMHs [17, 43]. Second, the microembolism may be another mechanism. Blood flow alteration and local turbulence at the site of tortuosity can damage the vascular endothelium and then result in the formation of the microemboli which will occlude the distal small blood vessels [45]. Furthermore, arterial tortuosity and WMHs may have some shared risk factors, such as age and hypertension. Our study also showed that age was significantly associated with the severity of WMHs, which is consistent with the previous studies [13, 14]. Interestingly, patients with severe WMHs had an increased tortuosity index of left carotid artery system in this study, instead of right anterior circulation. The reason for this result is not yet clear. We speculate that this phenomenon may be due to the different anatomical origins of bilateral CCA. The left CA directly from the aortic arch is affected by aortic arch pressure, while the right CA always originates from the brachiocephalic and is subjected to pressure from the ascending aortic blood flow [46]. In addition, left CA has a relatively longer pathway before entering the circle of Willis than the right side, so it more likely to cause hemodynamic changes.

There were several limitations in this study. First, since a case-control observational design causality of arterial tortuosity cannot be inferred, further validation of arterial tortuosity as a risk factor for AIS should be studied prospectively. Second, we included AIS patients identified as large arterial atherosclerosis. Arterial atherosclerosis or stenosis may also contribute to increased risk of stroke which could not be incorporated into the analysis. Third, we noticed that a case-control study reported the link between carotid artery tortuosity and connective tissue diseases [47], which reminded us a further prospective investigation into our topic exclusive of patients with connective tissue diseases as a confounding factor. Finally, not all patients underwent diffusion tensor imaging to quantify WMHs and susceptibility-weighted imaging to detect microbleed. Therefore, we could not evaluate other neuroimaging markers of CSVD.

In conclusion, our data suggest that increased extra- and intracranial large arteries tortuosity appear to be associated with AIS. Tortuosity of arteries, especially left EICA and VAD, may be an independent risk factor for WMH severity in AIS patients.

## Figures and Tables

**Table 1:** Correlation analysis of each arterial tortuosity with AIS.

Artery	Crude OR (95% CI)	*P* value	Adjusted OR (95% CI)	*P* value	AUC (95% CI)	*P* value
L.CA	1.109 (1.062-1.158)	<0.001	1.14 (1.068-1.217)	<0.001	0.709 (0.642-0.775)	<0.001
L.CCA	1.099 (1.041-1.159)	0.001	1.106 (1.031-1.187)	0.005	0.659 (0.586-0.733)	<0.001
L.ICA	1.049 (1.017-1.082)	0.002	1.044 (1-1.09)	0.048	0.665 (0.581-0.749)	0.001
L.EICA	1.137 (1.05-1.231)	0.002	1.307 (1.037-1.646)	0.023	0.79 (0.664-0.917)	<0.001
L.IICA	1.028 (0.994-1.063)	0.104	1.077 (1.014-1.143)	0.016	0.6 (0.435-0.766)	0.202
L.M1	1.028 (1.009-1.048)	0.005	1.026 (1.001-1.052)	0.043	0.644 (0.559-0.728)	0.001
R.CA	1.05 (1.012-1.088)	0.009	1.059 (1.007-1.113)	0.025	0.632 (0.56-0.704)	<0.001
R.CCA	1.06 (1.015-1.107)	0.009	1.066 (1.01-1.126)	0.02	0.647 (0.574-0.72)	<0.001
R.ICA	1.029 (0.999-1.061)	0.062	1.011 (0.97-1.052)	0.611	0.623 (0.537-0.708)	0.01
R.EICA	1.05 (0.995-1.108)	0.076	1.051 (0.985-1.122)	0.129	0.703 (0.565-0.841)	0.01
R.IICA	1.022 (0.993-1.052)	0.143	1.067 (1.012-1.124)	0.015	0.618 (0.457-0.779)	0.136
R.M1	1.014 (0.997-1.03)	0.1	1.029 (1.006, 1.053)	0.015	0.595 (0.505-0.685)	0.032
VAD	1.052 (1.018-1.087)	0.003	1.06 (1.009-1.113)	0.021	0.628 (0.555-0.701)	<0.001
BA	1.007 (0.977-1.038)	0.65	1.002 (0.967-1.039)	0.903	0.523 (0.446-0.6)	0.555

The adjusted odd ratio and *P* value represent the results of multivariate logistic regression analysis. Variables entered into analysis include age, male, hypertension, diabetes, hyperlipidemia, smoking, alcohol abuse, LDL-C, C-reactive protein, homocysteine, fibrinogen, and proteinuria. AUC: area under the receiver operating characteristic curve; CI: confidence interval; OR: odds ratio.

**Table 2:** Baseline characteristics of AIS patients and control subjects.

	AIS patients (*n* = 166)	Control subjects (*n* = 83)	*P* value
Age (year), mean ± SD	56.98 ± 0.83	55.86 ± 1.28	0.447
Male, *n* (%)	115 (69.3)	56 (67.5)	0.772
Medical history, *n* (%)			
Hypertension	103 (62.0)	36 (43.3)	0.005
Diabetes	31 (18.7)	13 (15.7)	0.557
Hyperlipidemia	12 (7.2)	10 (12.0)	0.207
Smoking	84 (50.6)	38 (45.8)	0.473
Alcohol abuse	61 (36.7)	27 (32.5)	0.512
Laboratory findings, median (IQR)			
Total cholesterol	4.1 (3.5-4.7)	4.1 (3.4-4.9)	0.909
Triglycerides	1.3 (1-1.7)	1.4 (1-1.7)	0.823
LDL-C	2.6 (2-3.1)	2.4 (1.8-3.2)	0.917
HDL-C	1 (0.8-1.2)	1 (0.8-1.2)	0.43
Serum creatinine	71 (61-82.8)	68 (59-77.5)	0.164
Uric acid	314.9 (259.8-365.9)	308.6 (248.1-386.2)	0.911
C-reactive protein	2.3 (1.1-5.4)	1 (0.6-2.4)	<0.001
Homocysteine	13.9 (11.6-19.2)	11.2 (8.7-16.7)	0.002
Fibrinogen	3.3 (2.9-4)	3 (2.6-3.6)	0.016
Proteinuria, *n* (%)	33 (19.9)	6 (7.2)	0.01
White matter hyperintensities, *n* (%)			0.003
Fazekas 0	15 (9)	17 (20.5)	
Fazekas 1	79 (47.6)	48 (57.8)	
Fazekas 2	46 (27.7)	11 (13.3)	
Fazekas 3	26 (15.7)	7 (8.4)	

AIS: acute ischemic stroke; HDL-C: high-density lipoprotein cholesterol; LDL-C: low-density lipoprotein cholesterol.

## Data Availability

The data that support the findings of this study are available from the corresponding author upon reasonable request.

## Supplementary Materials

Supplementary Materials Supplementary Figure 1: flow diagram of the study design. Supplementary Figure 2: measurement of arterial tortuosity by location from CTA. Supplementary Table 1: arterial tortuosity of patients with acute ischemic stroke and control subjects. Supplementary Table 2: baseline characteristics of severity of white matter hyperintensities in patients with acute ischemic stroke. Supplementary Table 3: arterial tortuosity by severity of white matter hyperintensities in patients with acute ischemic stroke. Supplementary Table 4: correlation analysis of each arterial tortuosity with WMH severity in AIS patients. Checklist of items that should be included in reports of observational studies.
