# Supplementary material for: Arterial Tortuosity and Its Correlation with White Matter Hyperintensities in Acute Ischemic Stroke
Source: Neural Plast. 2022 Mar 24;2022:4280410. doi: 10.1155/2022/4280410 (PMC8970938; doi:10.1155/2022/4280410)
Supplement: Supplementary Materials — Supplementary Figure 1: flow diagram of the study design. Supplementary Figure 2: measurement of arterial tortuosity by location from CTA. Supplementary Table 1: arterial tortuosity of patients with acute ischemic stroke and control subjects. Supplementary Table 2: baseline characteristics of severity of white matter hyperintensities in patients with acute ischemic stroke. Supplementary Table 3: arterial tortuosity by severity of white matter hyperintensities in patients with acute ischemic stroke. Supplementary Table 4: correlation analysis of each arterial tortuosity with WMH severity in AIS patients. Checklist of items that should be included in reports of observational studies. [file 4280410.f1.zip › 4280410.f1/SUPPLEMENTAL MATERIAL.docx]

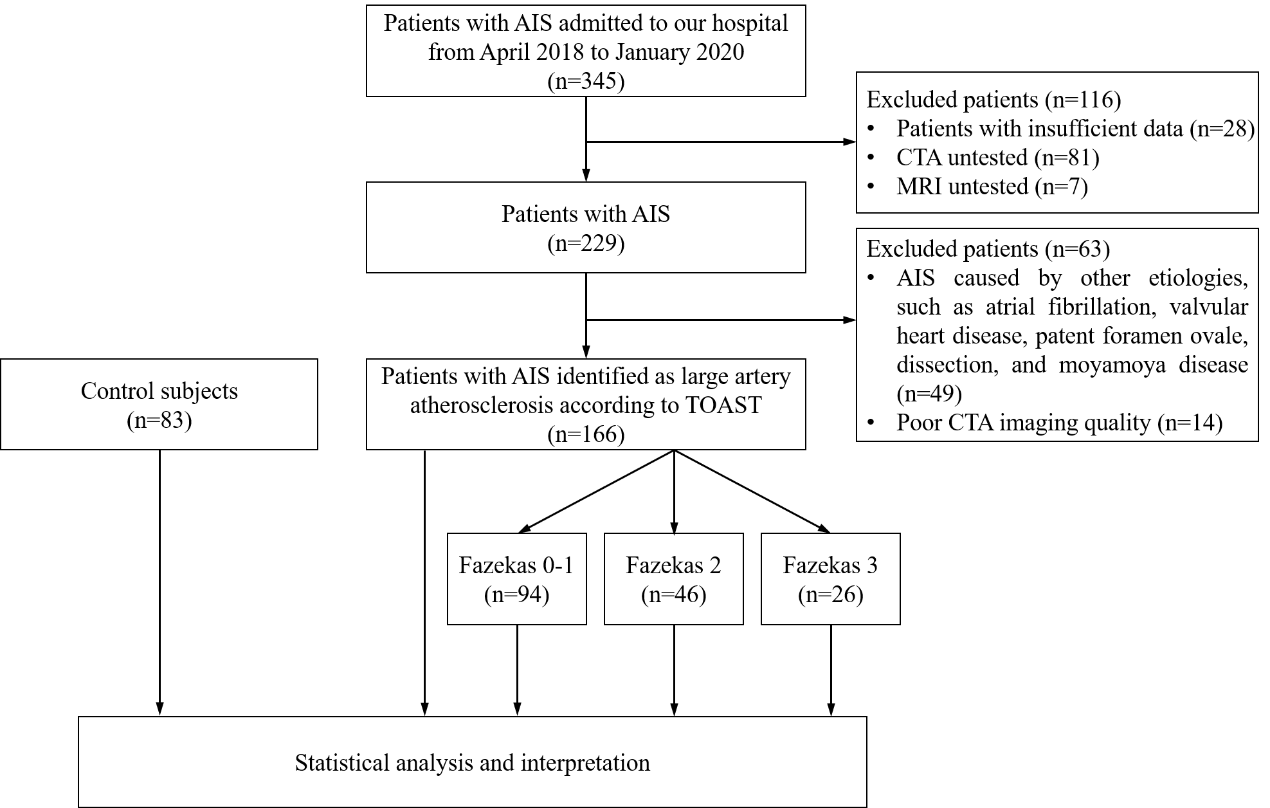


**Supplementary Figure 1**. Flow diagram of the study design. AIS patients were scored their WMHs using the visual rating scale proposed by Fazekas grades ranging from 0 to 3. Patients were divided into three groups according to the Fazekas grades: none to mild (Fazekas 0-1), moderate (Fazekas 2), severe (Fazekas 3).


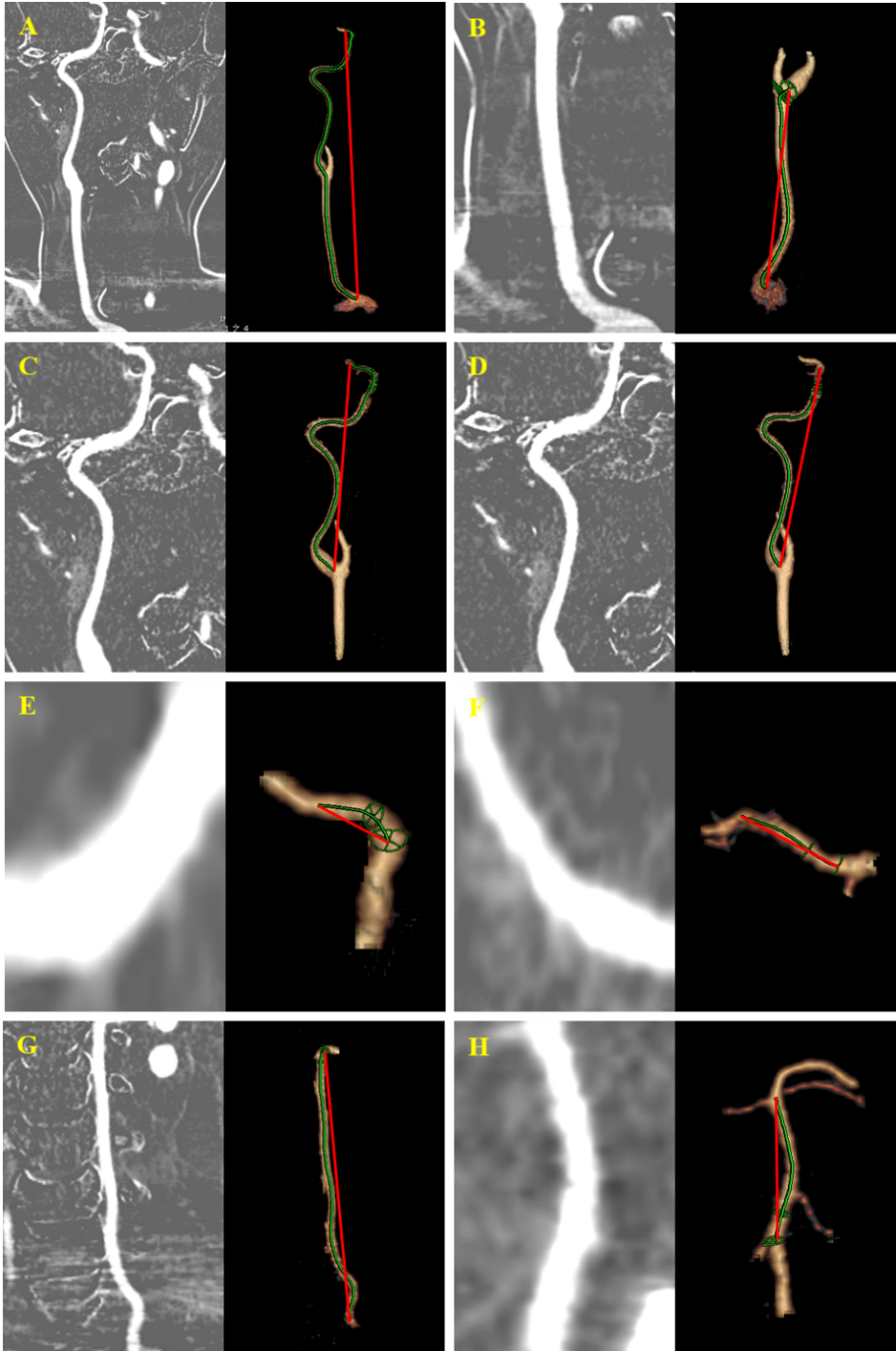


**Supplementary Figure 2**. Measurement of arterial tortuosity by location from CTA. Multiplanar reformation (left panel) and three-dimensional reconstructed imaging (right panel) are shown for arterial segment. The straight length (red line) and actual length (green line) are measured in three-dimensional CTA. (A) show right CA from the bifurcation point of the subclavian artery to the MCA-ACA bifurcation point. (B) show right CCA from the bifurcation point of the subclavian artery to the ICA-ECA bifurcation point. (C) show right ICA from the bifurcation point of CCA to MCA-ACA bifurcation point. (D) show right EICA from the bifurcation point of CCA to exocranial ostium of carotid canal. (E) show right IICA from exocranial ostium of carotid canal to ICA terminus. (F) show right first segment of MCA from the bifurcation point of ICA to the bifurcation point of branches. (G) show VAD from the bifurcation point of the subclavian artery to the vertebrobasilar junction. (H) show basilar artery from the vertebrobasilar junction to terminus. ACA, anterior cerebral artery; CA, carotid artery; CCA, common carotid artery; ECA, external carotid artery; EICA, extracranial internal carotid artery; ICA, internal carotid artery; IICA, intracranial internal carotid artery; MCA, middle cerebral artery; VAD, vertebral artery dominance.

**Supplementary Table 1**. Arterial tortuosity of patients with acute ischemic stroke and control subjects

| Tortuosity index (median, IQR) | AIS patients  (n=166) | Control subjects  (n=83) | *P* value |
| --- | --- | --- | --- |
| L.CA | 27.8 (22.8-34.4) | 22.1 (19-25.8) | <0.001 |
| L.CCA | 7.5 (5-12.3) | 5.2 (2.5-8.9) | <0.001 |
| L.ICA | 42.6 (34.9-51.4) | 36.5 (30.9-43.4) | 0.001 |
| L.EICA | 22.7 (11.9-33.4) | 11.6 (7.4-14.7) | <0.001 |
| L.IICA | 69.7 (50.7-84.5) | 61.1 (54.6-68.3) | 0.202 |
| L.M1 | 33.9 (25.3-46.4) | 26.4 (16.2-36.4) | 0.001 |
| R.CA | 27.5 (22-32.9) | 24.1 (20.8-28.2) | 0.001 |
| R.CCA | 6.8 (4.1-12) | 4.4 (2.2-7.9) | <0.001 |
| R.ICA | 40 (32.5-49.1) | 34.1 (30.7-40.5) | 0.01 |
| R.EICA | 14.8 (9.7-21.1) | 6.2 (3.5-15.5) | 0.01 |
| R.IICA | 76.3 (53.5-90) | 63.1 (54.1-71) | 0.136 |
| R.M1 | 28.8 (20.1-41.8) | 22.9 (12.3-38.7) | 0.032 |
| VAD | 19.4 (14.6-26.1) | 16.2 (11.7-21.1) | 0.001 |
| BA | 7.4 (4.1-12.4) | 6.9 (4.5-11.9) | 0.555 |

AIS, acute ischemic stroke; BA, Basilar artery; CA, carotid artery; CCA, common carotid artery; CI, confidence interval; EICA, extracranial internal carotid artery; ICA, internal carotid artery; IICA, intracranial internal carotid artery; L, left; M1, first segment of middle cerebral artery; R, right; VAD, vertebral artery dominance.

**Supplementary Table 2**. Baseline characteristics of severity of white matter hyperintensities in patients with acute ischemic stroke.

|  | Fazekas 0-1  (n=94) | Fazekas 2  (n=46) | Fazekas 3  (n=26) | *P* value |
| --- | --- | --- | --- | --- |
| Age, y, mean ± SD | 53.2±1.1 | 60.2±1.2 | 65.0±1.8 | <0.001 |
| Male, n (%) | 69 (73.4) | 32 (69.6) | 14 (53.8) | 0.16 |
| Medical history, n (%) | | | | |
| Hypertension | 52 (55.3) | 34 (73.9) | 17 (65.4) | 0.096 |
| Diabetes | 17 (18.1) | 6 (13.0) | 8 (30.8) | 0.196 |
| Hyperlipidemia | 4 (4.3) | 3 (6.5) | 5 (19.2) | 0.066 |
| Smoking | 46 (48.9) | 28 (60.9) | 10 (38.5) | 0.167 |
| Alcohol abuse | 37 (39.4) | 15 (32.6) | 9 (34.6) | 0.717 |
| Laboratory findings, median (IQR) | | | | |
| Total cholesterol | 4.3 (3.6-4.9) | 3.9 (3.3-4.3) | 4.2 (3.1-4.6) | 0.243 |
| Triglycerides | 1.4 (1-1.9) | 1.2 (1-1.6) | 1.3 (0.9-2) | 0.447 |
| LDL-C | 2.7 (2.0-3.3) | 2.5 (1.9-2.9) | 2.5 (1.6-2.9) | 0.674 |
| HDL-C | 1.0 (0.8-1.2) | 1.0 (0.8-1.1) | 1.1 (0.8-1.3) | 0.651 |
| Serum creatinine | 71 (65.3-83) | 72 (60.1-83.3) | 64.5 (53.3-82) | 0.142 |
| Uric acid | 318 (263-365) | 318 (265-384) | 291 (244-346) | 0.63 |
| C-reactive protein | 2.7 (1.1-5.5) | 1.8 (0.9-6.3) | 2.1 (1.1-4.9) | 0.488 |
| Homocysteine | 13.5 (10.9-18.8) | 14.4 (11.7-19.6) | 14.7 (12-20.8) | 0.627 |
| Fibrinogen | 3.2 (2.9-3.9) | 3.5 (3.1-4.2) | 3.4 (3.0-3.8) | 0.761 |
| Proteinuria, n (%) | 19 (20.2) | 12 (26.1) | 2 (7.7) | 0.17 |

HDL-C, high-density lipoprotein cholesterol; LDL-C, low-density lipoprotein cholesterol.

**Supplementary Table 3**. Arterial tortuosity by severity of white matter hyperintensities in patients with acute ischemic stroke

| Tortuosity index (median, IQR) | Fazekas 0-1 | Fazekas 2 | Fazekas 3 | *P* value |
| --- | --- | --- | --- | --- |
| L.CA | 26.3 (21.6-33.5) | 28.4 (23.2-33.8) | 31.7 (26.2-44.6) | 0.005 |
| L.CCA | 6.9 (4.5-11.1) | 6.8 (5.3-10.3) | 13.5 (7.8-20.1) | 0.003 |
| L.ICA | 42.6 (33.7-50.3) | 43.9 (36.2-55.6) | 41.8 (35.7-57.6) | 0.706 |
| L.EICA | 15.3 (10.3-26.7) | 17.4 (10.3-31.0) | 33.7 (30.5-54.3) | 0.027 |
| L.IICA | 68.6 (55.2-82.1) | 54.2 (45.9-85.2) | 89.1 (58.1-105.9) | 0.144 |
| L.M1 | 35.1 (24.5-50.8) | 32.1 (26.8-43.1) | 28.9 (24.5-45.7) | 0.812 |
| R.CA | 26.5 (21.8-31.2) | 28.8 (23.6, 33.0) | 31.1 (21.6-36.3) | 0.073 |
| R.CCA | 6.3 (4.2-10.7) | 7.6 (3.6-13.0) | 8.5 (5.2-19.9) | 0.251 |
| R.ICA | 40.2 (32.2-46.3) | 38.8 (32.9-50.8) | 41.4 (30.6-53.9) | 0.691 |
| R.EICA | 14.8 (9.7-21.2) | 10.7 (6.6-16.9) | 19.0 (14.6-38.3) | 0.129 |
| R.IICA | 76.8 (52.4-78.2) | 69.5 (49.8-82.1) | 83.8 (53.3-111.1) | 0.537 |
| R.M1 | 28.6 (19.6-42.4) | 31.0 (22.2-45.0) | 27.0 (19.1-37.1) | 0.682 |
| VAD | 17.8 (14.1-24.0) | 20.7 (14.7-25.3) | 27.5 (16.7-37.8) | 0.001 |
| BA | 9.5 (4.9-13.6) | 6.3 (3.5-8.6) | 7.8 (3.1-15.8) | 0.130 |

**Supplementary Table 4**. Correlation analysis of each arterial tortuosity with WMHs severity in AIS patients

| Artery | Fazekas 0-1 | Fazekas 2 | | Fazekas 3 | |
| --- | --- | --- | --- | --- | --- |
|  |  | OR (95%CI) | *P* value | OR (95%CI) | *P* value |
| L.CA | 1 [Reference] | 0.983 (0.936-1.033) | 0.5 | 1.056 (0.998-1.118) | 0.058 |
| L.CCA | 1 [Reference] | 0.976 (0.919-1.037) | 0.437 | 1.046 (0.985-1.112) | 0.145 |
| L.ICA | 1 [Reference] | 1.001 (0.97-1.033) | 0.959 | 1.005 (0.962-1.05) | 0.835 |
| L.EICA | 1 [Reference] | 1.02 (0.927-1.122) | 0.688 | 1.119 (1.006-1.244) | 0.039 |
| L.IICA | 1 [Reference] | 0.974 (0.912-1.041) | 0.444 | 1.067 (0.983-1.158) | 0.119 |
| L.M1 | 1 [Reference] | 0.986 (0.965-1.008) | 0.215 | 0.988 (0.961-1.017) | 0.422 |
| R.CA | 1 [Reference] | 0.993 (0.948-1.04) | 0.765 | 1.019 (0.966-1.076) | 0.483 |
| R.CCA | 1 [Reference] | 1.003 (0.957-1.051) | 0.907 | 1.033 (0.98-1.088) | 0.227 |
| R.ICA | 1 [Reference] | 0.994 (0.96-1.03) | 0.75 | 0.998 (0.948-1.05) | 0.926 |
| R.EICA | 1 [Reference] | 0.915 (0.73-1.146) | 0.438 | 1.117 (0.925-1.349) | 0.25 |
| R.IICA | 1 [Reference] | 0.973 (0.915-1.034) | 0.37 | 1.022 (0.965-1.081) | 0.458 |
| R.M1 | 1 [Reference] | 1.009 (0.989-1.029) | 0.372 | 1 (0.966-1.036) | 0.986 |
| VAD | 1 [Reference] | 0.994 (0.953-1.037) | 0.781 | 1.056 (1.008-1.107) | 0.022 |
| BA | 1 [Reference] | 0.947 (0.892-1.006) | 0.076 | 1.004 (0.954-1.057) | 0.877 |

The adjusted odd ratio and *P* value represent the results of multivariate logistic regression analysis. Variables entered into analysis including: age, male, and hypertension. CI indicates confidence interval, OR, odds ratio.
